# Effects of spirulina as a functional ingredient in arsenic-induced broiler diet on growth performance and hematobiochemical parameters

**DOI:** 10.5455/javar.2022.i619

**Published:** 2022-09-30

**Authors:** Md. Mowdudul Hasan Talha, Md. Anwar Hossain, Md. Aktaruzzaman, Md. Siddiqul Islam, Akash Khasnobish, Md. Rashedunnabi Akanda

**Affiliations:** 1 Department of Pharmacology and Toxicology, Sylhet Agricultural University, Sylhet, Bangladesh; 2 Faculty of Veterinary, Animal and Biomedical Sciences, Sylhet Agricultural University, Sylhet, Bangladesh

**Keywords:** Arsenic, body weight, broiler, hematobiochemical, spirulina

## Abstract

**Objectives:**

The purpose of this research was to look into the impacts after the implication of feeding broiler chickens with spirulina in arsenic-incited toxicities.

**Materials and Methods:**

Birds (*n =* 125) were distributed equally (*n =* 25) into four groups treated (*T*_1_, *T*_2_, *T*_3_, *T*_4_) and a group controlled, *T*_0_ (normal feed and water without supplement), the group taking in arsenic trioxide (100 mg/l)-induced diet (*T*_1_), and the groups *T*_2_, *T*_3_, and *T*_4_ (feed supplemented with 50, 100, and 200 mg/l of spirulina along with Arsenic Trioxide, respectively). The body weight and hematobiochemical parameters were recorded every 7 days.

**Results:**

Different growth development indicators, e.g., body weight, feed intake ratio, feed conversion ratio, depression, and skin lesions, were weak in arsenic trioxide groups and upstanding in the arsenic plus spirulina group. Over and above, the lack of body weight gain in chicken (2.7%–13.00%) in the arsenic-introduced groups given spirulina (*T*_2_, *T*_3_, and *T*_4_) overtook the mere groups exposed to arsenic, where the lack of weight gain was optimum (54.90%). Thereafter, in arsenic-instituted groups given spirulina (*T*_2_, *T*_3_, and *T*_4_), the drop in total erythrocyte count, total leukocyte count, hemoglobin, and packed cell volume values became less notable than in arsenic pollutant groups (*T*_1_, *p <* 0.01). Two measurable factors (serum glutamate pyruvate transaminase and serum glutamic oxaloacetic transaminase) were substantially (*p <* 0.01) raised in the group (*T*_1_) treated with arsenic, but in the arsenic-induced groups (*T*_2_, *T*_3_, and *T*_4_) treated with spirulina, they were elevated less.

**Conclusion:**

This study demonstrates that arsenic is a threat to poultry. However, spirulina may be advantageous for alleviating the effects of arsenic in poultry.

## Introduction

Bangladesh is an agricultural-based, densely populated country where livestock is one of the major sectors, contributing 1.43% of the total GDP [1]. Among livestock industries, the poultry industry stands out because it provides a foundation for producing animal proteins most efficiently and cost-effectively possible in the shortest amount of time while also providing a diverse range of job opportunities [2,3]. One of the primary causes that harm the food web is arsenic, and the country is inclined significantly toward the occurrence of arsenic-caused maladies [4]. The most toxic forms of arsenic are inorganic [5]. Through contaminated drinking water and food, a greater portion of the population is exposed to inorganic arsenic [6].

Under Bangladeshi circumstances, the majority of poultry farms count on a perfunctory water source that is more contaminated with arsenic in comparison to deep well water [7]. Providing broilers with arsenic-contaminated feed and water leads to the deposition of arsenic loads in their body muscles and droppings [8]. Cooking arsenic-intoxicated meat, however, may result in more arsenic-rich toxic by-products for customers [9]. Arsenic can accumulate in broiler flesh and harm consumers if the permissible levels in broiler feed and water are not adequately followed. According to clinical studies, algae with a high mineral and vitamin concentration may affect people with heavy metal toxicity [10].

Spirulina, a microscopic blue-green algae, is a good candidate as an in-feed antibiotic substitute for broilers and has the property of detoxifying metal toxicities from the blood [11]. Spirulina reduces tissue accumulation of mercury and other harmful metals [12]. It has been discovered that adding less than 1% spirulina to chicken diets strengthens defensive systems by boosting microbial killing, antigen processing, and T-cell activation [13]. Henceforth, being a cyanobacterium, it has been profitably grown for many years because of its rich nutritional value, which includes amino acids, protein, vitamins, minerals, fatty acids, and β-carotene [10,14].

In Bangladesh, there’s very limited information on the level of arsenic in broilers generated by birds given feed and drinking water that contain arsenic [8]. The effects of arsenic buildup in broiler chickens following short-term high-dose exposure remain unknown. The current study was done under these conditions to see what effect eating spirulina had on body weight gain and biochemical components in the blood of broilers that had been poisoned by arsenic.

## Materials and Methods

### Ethical approval

An authorization form was issued by the farm owner in partaking. While taking blood samples, every effort was made to minimize the stress and discomfort of the broiler chickens. The People‘s Republic of Bangladesh‘s Animal Welfare Act 2019 and the Cruelty to Animals Act of 1920 were followed when handling animals. The Sylhet Agricultural University‘s Ethical Committee in Bangladesh approved the specific experimental plan. The ethical number that was authorized was #AUP2019004.

### Study design

A total of 125 Cobb-500 broiler chicks were purchased from Kazi Farms, Sylhet. They were fed with standard broiler starter, grower, and finisher ration (CP Bangladesh Company Ltd.) based on their nutritional requirements. Initial body weight did not differ between the groups. Chicks were placed into five equal groups (*n =* 25) and given the labels *T*_0_, *T*_1_, *T*_2_, *T*_3_, and *T*_4_ after assimilating for 7 days. *T*_0_ served as a reference for whether *T*_1_ was given arsenic trioxide (Loba Chemicals Co. Bombay, India) at 100 mg/l. *T*_2_ was provided with arsenic trioxide at 100 mg/l plus spirulina (Eskalina^®^, Eskayef Pharmaceuticals Ltd.) at 50 mg/l. *T*_3_ was treated with arsenic trioxide at 100 mg/l plus spirulina at 100 mg/l. *T*_4_ was fed with arsenic trioxide at 100 mg/l plus spirulina at 200 mg/l. All the treatments were given with regular recommended feed and drinking water ad. lib.

The following study parameters were taken on days 7, 14, 21, and 28:

- Clinical signs and body weight
- Hematological parameters, i.e., total erythrocyte count (TEC), total leukocyte count (TLC), packed cell volume (PCV), and hemoglobin (Hb)
- Biochemical parameters, i.e., serum glutamate pyruvate transaminase (SGPT) and serum glutamic oxaloacetic transaminase (SGOT).

### Clinical signs and body weight

Both the control and treated bird groups were looked at cautiously for rising of any gross lesion during the study period. Until day 28, the birds‘ body weight was assessed at intervals of 7 days (7, 14, 21, and 28). The body weight of each bird was taken before feeding in the morning and expressed in grams (gm) with the help of an electric balance.

### Blood collection

Aseptically, 3–4 ml of blood was taken from the wing vein. Each sample was immediately divided by dispensing 1 ml of blood to a clean, dried test tube containing anticoagulant (ethylenediaminetetraacetic acid; EDTA) and the other 1.5 ml of blood without anticoagulant. This led to collecting the serum for biochemical studies.

### Hematological examination

MythicTM18 was used to determine the hematological parameters, a completely automated hematology analyzer that does a complete blood count. About 1 ml EDTA blood sample was put in the instrument‘s aspirator, and the blood sample was aspirated. The data were shown on LCD in less than a minute, printed on the printer, and recorded in the resident memory.

### Biochemical examination

Blood sera biochemical parameters SGOT or aspartate aminotransferase (AST) and SGPT or alanine transferase (ALT) were detected from the serum using the specific test kit (Randox) and semi-auto biochemical analyzer.

Result: The ALT/AST activity or SGPT/SGOT value of the provided sample was given in U/l.

### Statistical analysis of experimental data

The data gathered throughout the trial were statistically analyzed using completely randomized design and with the help of the STATA-13 software and Duncan’s multiple range test (DMRT) for ranging [15].

## Results and Discussion

### Clinical signs of arsenic-induced toxicities in broiler chickens

Throughout the trial, all of the control birds were normal and exhibited no indications of toxicity. For up to 14 days, chickens in group *T*_1_ (only arsenic trioxide) ran smoothly. Birds in the arsenic trioxide group revealed a rapid start of restlessness, lower feed intake, dullness, and ruffled feathers after 14 days. The skin lesion progressively developed in the legs after 28 days.

Up to 28 days of arsenic trioxide feeding, chickens in groups *T*_2_ (arsenic trioxide plus spirulina at 50 mg/l), *T*_3_ (arsenic trioxide plus spirulina at 100 mg/l), and *T*_4_ (arsenic trioxide plus spirulina at 200 mg/l) remained normal. Throughout the study, the birds in groups *T*_2_, *T*_3_, and *T*_4_ had no skin lesions. Similar toxic symptoms were seen in birds following arsenic trioxide feeding and after arsenic trioxide and spirulina feeding at different dosages in the proposed investigation. Most of the indications shown in *T*_1_ (save for arsenic trioxide) were also seen in the other three groups (*T*_2_, *T*_3_, and *T*_4_), although in a milder form. Lesions on the skin that were especially noticeable in the *T*_1_ group (only arsenic trioxide) were not present in the other three groups that were given spirulina. This suggests that spirulina protects against skin lesions caused by arsenic trioxide.

Islam et al. [16] spotted that arsenic-treated rats exhibited adverse effects of excitement, irritability, loss of appetite, fringed hair coat, and skin problems in all body parts, particularly on the tail. Noxious indications in *T*_1_ (arsenic treated) are partially consistent with his postulation. Ramanathan [17] found reduced meal intake and a ruffled hair coat in arsenic-treated rats.

The aforementioned findings are also consistent with Smith et al. [18], Lasky et al. [19], and Mitra et al. [20]. A diet lacking fiber, calcium, folate, and animal protein might increase vulnerability to skin lesions brought on by arsenic [20]. According to Lasky et al. [19], arsenic, in its inorganic forms, is categorized as a carcinogen, with chronic exposure (10–40 gm/d) leading to skin, lung, and bladder cancers.

### Effects of spirulina on body weight in arsenic-induced toxicities in broiler chickens

The body weight of birds in each group was measured 7 days after they were given arsenic trioxide and spirulina (Table 1). At the end of 28 days, the mass weight growth of chickens in the control group (*T*_0_) was the greatest (1,609.75 gm), whereas the body weight gains of arsenic-treated birds (*T*_1_) were the lowest (209.12, 317.75, 431.75, and 729.5 gm).

Despite the reality of their growing age, most birds gained weight gradually, although the body weight gain rate in group *T*_1_ was significantly lower (*p <* 0.01). Compared to the control group, the birds with arsenic treatment group *T*_1_ had the highest percentage of not increased body weight (54.90%). Similar populations treated, such as arsenic-induced spirulina given groups *T*_2_, *T*_3_, and *T*_4_, had body weight reductions of 13.00%, 6.60%, and 2.70%, which were lower than the only arsenic-induced group.

In this study, arsenic has been shown to diminish the developing pattern of body weight in chickens. In experimentally produced arsenic toxicosis in ducks, Islam et al. [8] found that arsenic substantially (*p <* 0.01) decreased body weight. Islam et al. [8] found that arsenic-treated chickens in India had a lower body weight [8]. Similarly, the effects of sequential heavy metal intake in rats were studied [21]. Administration of both metals, cadmium and arsenic, decreased weight gain more than administration of either metal alone, which was true even after accounting for changes in food intake. Islam et al. [16] found remarkably (*p <* 0.01) decreased weight gain in arsenic-treated rats, which is appropriate for the current findings. Sharma et al. [22] found that arsenic-treated Swiss albino mice showed lower body weight.

**Table 1.** Body weight (gm) of control, arsenic-induced, and arsenic-and spirulina-treated chickens.

Treatment	Day 7	Day 14	Day 21	Day 28	% Not gained
Control (*T*_0_)	207.62 ± 6.86	505.87^b^ ± 9.77	1,022^c^ ± 27.30	1,609.75^c^ ± 35.0	
Arsenic trioxide at 100 mg/l (*T*_1_)	209.12 ± 6.55	317.75^c^ ± 6.44	431.75^d^ ± 32.8	729.5^d^ ± 40.80	54.90
Arsenic trioxide at 100 mg/l plus spirulina at 50 mg/l (*T*_2_)	202.62 ± 5.72	470.25^a^ ± 19.5	897.12^b^ ± 22.2	1,395.25^b^ ± 49.5	13.00
Arsenic trioxide at 100 mg/l plus spirulina at 100 mg/l (*T*_3_)	204 ± 4.07	513.25^a^ ± 7.39	980.37^b^ ± 26.3	1,504.25^ab^ ± 37.90	6.60
Arsenic trioxide at 100 mg/l plus spirulina at 200 mg/l (*T*_4_)	206.5 ± 5.87	516.25^a^ ± 14.8	1,001.5^b^ ± 30.50	1,566^ab^ ± 60.00	2.70

Figure in mean.

SE: Standard error.

According to DMRT, column figures with or without superscripts do not change substantially.

Dates were estimated at the 99% level of significance (*p <* 0.01).

### Hematological parameters

### Total erythrocyte count

Table 2 shows the impact of daily administration of arsenic trioxide alone and in combination with spirulina at different dosages in drinking water on TEC in chickens for 28 days. In comparison to the control group (*T*_0_), the TEC values in chickens were substantially lower (*p <* 0.01) in the arsenic trioxide-fed group (*T*_1_). The reduction in TEC values in the other three groups (*T*_2_, *T*_3_, and *T*_4_), i.e., combined arsenic and spirulina treatment, was 19.20%, 8.00%, and 3.20%, respectively, which was lower than the arsenic-treated groups.

### Total leucocyte count

Tables 3 and 4 show the effects of the daily administration of arsenic trioxide alone and in combination with spirulina in drinking water for 28 days. The TEC readings in chickens became substantially lower (*p <* 0.01) in the arsenic trioxide-fed group (*T*_1_) than in the control group (*T*_0_). The reduction of TLC values in the other three groups (*T*_2_, *T*_3_, and *T*_4_), i.e., combined arsenic and spirulina treatment, was 3.60%, 2.90%, and 0.60%, respectively, which was lower than the arsenic-treated groups.

### Hemoglobin

Table 4 shows the effects of combining arsenic trioxide with spirulina at various dosages in drinking water on Hb levels in chickens. Hb levels in chickens were reduced remarkably (*p <* 0.01) by 15.10% in the arsenic-given group, similar to TEC (*T*_1_). The Hb value decreased to 8.60% and 5.40 % in the arsenic plus spirulina treaded group (*T*_2_).

**Table 2.** TEC (million/µl) of control, arsenic-induced, and arsenic-and spirulina-treated chickens.

Treatment	Day 7	Day 14	Day 21	Day 28	% Decreased
Control (*T*_0_)	2.59 ± 0.34	2.80^a^ ± 0.18	2.95^a^ ± 0.08	3.02^a^ ± 0.29	
Arsenic trioxide at 100 mg/l (*T*_1_)	2.42 ± 0.30	2.11a ± 0.95	2.27^ab^ ± 0.53	2.37^a^ ± 0.15	20.8
Arsenic trioxide at 100 mg/l plus spirulina at 50 mg/l (*T*_2_)	2.50 ± 0.25	2.20a ± 0.44	2.33^ab^ ± 0.30	2.44^abc^ ± 0.17	19.2
Arsenic trioxide at 100 mg/l plus spirulina at 100 mg/l (*T*_3_)	2.55 ± 0.20	2.35a ± 0.87	2.42^d^ ± 0.29	2.51^bc^ ± 0.29	8.00
Arsenic trioxide at 100 mg/l plus spirulina at 200 mg/l (*T*_4_)	2.59 ± 0.34	2.80^a^ ± 0.18	2.95^a^ ± 0.08	3.02^a^ ± 0.29	3.20

Figure in mean.

SE: Standard error.

According to DMRT, column figures with or without superscripts do not change substantially.

Dates were estimated at the 99% level of significance (*p <* 0.01).

**Table 3.** TLC (Th/cumm) of control, arsenic-induced, and arsenic-and spirulina-treated chickens.

Treatment	Day 7	Day 14	Day 21	Day 28	% Decreased
Control (T_0_)	121.32 ± 0.33	119.11^a^ ± 0.18	116.76^a^ ± 0.08	116.98^a^ ± 0.29	
Arsenic trioxide at 100 mg/l (*T*_1_)	121.21 ± 0.30	113.78^b^ ± 0.95	112.74^c^ ± 0.53	117.63^a^ ± 0.15	3.60
Arsenic trioxide at 100 mg/l plus spirulina at 50 mg/l (*T*_2_)	121.27 ± 0.25	113.37^b^ ± 0.44	114.39^b^ ± 0.30	119.76^c^ ± 0.17	2.90
Arsenic trioxide at 100 mg/l plus spirulina at 100 mg/l (*T*_3_)	121.55 ± 0.27	117.18^a^ ± 0.87	115.31^b^ ± 0.29	120.46^b^ ± 0.29	2.40
Arsenic trioxide at 100 mg/l plus spirulina at 200 mg/l (*T*_4_)	121.15 ± 0.28	119.1^a^ ± 0.40	117.43^a^ ± 0.42	121.25^b^ ± 0.13	0.60

Figure in mean.

SE: Standard error.

According to DMRT, column figures with or without superscripts do not change substantially.

Dates were estimated at the 99% level of significance (*p <* 0.01).

### Packed cell volume

Table 5 shows the effects of daily administration of merely arsenic trioxide and arsenic plus spirulina at different dosages in drinking water on PCV in chickens for 28 days. PCV levels in hens were reduced considerably (*p <* 0.01) by 28.80% in the arsenic treatment group (*T*_1_) as compared to the control group (*T*_0_).

Many studies have demonstrated that arsenic reduces hematological parameters. Islam et al. [16] showed that TEC, TLC Hb, and PCV values were substantially (*p <* 0.01) decreased in arsenic-treated rats. In arsenic-treated ducks, Islam et al. [8] found a substantial (*p <* 0.01) drop in TEC, Hb, and PCV, as well as a significant (*p <* 0.01) rise in ESR. Islam et al. [8] also reported a remarkable (*p <* 0.01) reduction in TEC, TLC, Hb concentration, and PCV values.

Mahaffey and Fowler [21], on the other hand, reported a rise in the quantity of RBC count in rats. Moreover, in that case, arsenic toxicity lowered Hb concentration and hematocrit levels in the rats, as shown in the current investigation. The inhibitory action of arsenic compounds on the hematopoietic system, which is liable for the variances in hematobiochemical parameters, might explain the shift in hematobiochemical values. Additionally, arsenic trioxide’s effects on bone marrow were held accountable for erythrocytopenia by Islam et al. [16]. In ducks, this study was comparable to Islam et al. [8].

### Biochemical parameters

### Serum gluemate pyruvate transaminase ALT

Table 6 shows the effects of daily administration of merely arsenic trioxide and arsenic plus spirulina in different dosages in drinking water on SGPT in chickens. As compared to the control, the SGPT values in chickens rose (79.90%) substantially (*p <* 0.01) in the group (*T*_1_) where arsenic trioxide was fed. However, SGPT values in the other three categories are growing (*T*_2_, *T*_3,_ and *T*_4_) viz. 61.90%, 43.90%, and 29.60%, less than the *T*_1_ group.

**Table 4.** Hb (gm %) of control, arsenic-induced, and arsenic-and spirulina-treated chickens.

Treatment	Day 7	Day 14	Day 21	Day 28	% Decreased
Control (*T*_0_)	11.40 ± 0.10	11.51^a^ ± 0.11	11.55^a^ ± 0.15	12.62^a^ ± 0.12	
Arsenic trioxide at 100 mg/l (*T*_1_)	11.22 ± 0.11	10.50^ab^ ± 0.23	9.90^a^ ± 0.05	9.86^a^ ± 0.05	15.10
Arsenic trioxide at 100 mg/l plus spirulina at 50 mg/l (*T*_2_)	11.15 ± 0.08	10.80^a^ ± 0.28	10.71^a^ ± 0.25	10.60^a^ ± 0.15	13.70
Arsenic trioxide at 100 mg/l plus spirulina at 100 mg/l (*T*_3_)	11.30 ± 0.10	10.90^a^ ± 0.57	10.91^ab^ ± 0.24	11.56^ab^ ± 0.30	8.60
Arsenic trioxide at 100 mg/l plus spirulina at 200 mg/l (*T*_4_)	11.51 ± 0.09	11.12^ab^ ± 0.42	11.10^b^ ± 0.29	12.98^b^ ± 0.40	5.40

Figure in mean.

SE: Standard error.

According to DMRT, column figures with or without superscripts do not change substantially.

Dates were estimated at the 99% level of significance (*p <* 0.01).

**Table 5.** PCV (%) of control, arsenic-induced, and arsenic-and spirulina-treated chickens.

Treatment	Day 7	Day 14	Day 21	Day 28	% Decreased
Control (*T*_0_)	34.50 ± 0.33	40.58^a^ ± 0.17	40.47^a^ ± 0.14	43.06^a^ ± 0.52	
Arsenic trioxide at 100 mg/l (*T*_1_)	34.80 ± 0.23	30.33^a^ ± 0.73	30.06^a^ ± 0.08	30.88^a^ ± 0.58	28.80
Arsenic trioxide at 100 mg/l plus spirulina at 50 mg/l (*T*_2_)	34.88 ± 0.24	33.904^a^ ± 0.54	32.80^a^ ± 0.56	32.85^a^ ± 1.11	9.80
Arsenic trioxide at 100 mg/l plus spirulina at 100 mg/l (*T*_3_)	34.99 ± 0.22	34.10^a^ ± 1.05	33.50^a^ ± 1.03	33.41^a^ ± 1.33	7.60
Arsenic trioxide at 100 mg/l plus spirulina at 200 mg/l (*T*_4_)	32.46 ± 0.17	34.33^b^ ± 1.83	34.27^b^ ± 0.75	36.68^b^ ± 2.55	1.70

Figure in mean.

SE: Standard error.

According to DMRT, column figures with or without superscripts do not change substantially.

Dates were estimated at the 99% level of significance (*p <* 0.01).

### Serum glutamate oxaloacetate transaminaseAST

Table 7 shows the SGOT of chickens that were given arsenic trioxide alone or arsenic plus spirulina in different doses in their drinking water every day for 28 days.

SGOT levels in chickens were similarly raised substantially (*p <* 0.01) by 37.10% in the arsenic trioxide treatment group (*T*_1_) compared to the control group (*T*_0_). Nevertheless, in the *T*_2_, *T*_3_, and *T*_4_ groups, the values climbed to 28.30%, 23.50%, and 15.90%, lower than in the arsenic-treated group.

The blood levels of aminotransferase are significantly increased in animals susceptible to arsenic. However, the specific mechanism causing this elevation has yet to be determined. Several researchers have hypothesized that this effect is caused by cellular injury and/or enhanced plasma membrane permeability [23]. Furthermore, variables such as increased enzyme production and decreased enzyme degradation might be at play [24]. In arsenic-treated ducks, Islam et al. [23] found significantly (*p <* 0.01) higher SGPT and SGOT levels. In research published in 1999, Chiou et al. [25] discovered that ingesting arsenic raised the levels of SGPT, SGOT, ALP, and LDH in mice. Many authors have observed elevated biochemical markers due to arsenic poisoning, similar to the current findings [16,22,26]. As a result of arsenic poisoning, several authors found increased biochemical markers, which are comparable to this study [16,22,26]. The plasma chemistry of Nigerian ducks (*Anas platyrhynchos*) was determined to be 14 mature (50–80 weeks old) and 10 tenders (8–10 weeks old) being studied. According to the researchers, baby birds have far higher amounts of AST and ALT than adult birds [26].

Serum biochemical markers were considerably increased in this investigation, showing that arsenic trioxide produced some lesions or damage. The increase in all measures was greatest in the *T*_1_ group (fed arsenic alone). The other three groups (*T*_2_, *T*_3_, and *T*_4_) received spirulina in three different dosages coupled with arsenic trioxide and saw a lower rise in these biochemical markers.

**Table 6.** SGPT/ALT (U/l) of control, arsenic-induced, and arsenic-and spirulina-treated chickens.

Treatment	Day 7	Day 14	Day 21	Day 28	% Increased
Control (*T*_0_)	23.37 ± 0.75	22.00^b^ ± 0.59	25.62^a^ ± 0.62	23.62^b^ ± 0.49	
Arsenic trioxide at 100 mg/l (*T*_1_)	23.00 ± 0.73	27.28^c^ ± 0.80	30.22^b^ ± 0.49	33.24^c^ ± 0.52	79.90
Arsenic trioxide at 100 mg/l plus spirulina at 50 mg/l (*T*_2_)	22.75 ± 0.64	25.10^d^ ± 0.25	28.37^c^ ± 0.65	31.25^a^ ± 0.80	61.90
Arsenic trioxide at 100 mg/l plus spirulina at 100 mg/l (*T*_3_)	23.12 ± 0.44	23.23^a^ ± 0.42	27.23^d^ ± 0.58	29.62^a^ ± 0.75	43.90
Arsenic trioxide at 100 mg/l plus spirulina at 200 mg/l (*T*_4_)	23.25 ± 0.59	21.92^a^ ± 0.88	26.45^e^ ± 0.35	27.75^d^ ± 0.73	29.60

Figure in mean.

SE: Standard error.

According to DMRT, column figures with or without superscripts do not change substantially.

Dates were estimated at the 99% level of significance (*p <* 0.01).

**Table 7.** SGOT/AST (U/l) of control, arsenic-induced, and arsenic-and spirulina-treated chickens.

Treatment	Day 7	Day 14	Day 21	Day 28	% Increased
Control (*T*_0_)	209 ± 0.96	205^b^ ± 6.24	203.28^b^ ± 2.45	207.37^b^ ± 1.40	
Arsenic trioxide at 100 mg/l (*T*_1_)	207.5 ± 1.05	272.75^c^ ± 16.3	273.75^a^ ± 0.70	313.62^c^ ± 4.86	37.10
Arsenic trioxide at 100 mg/l plus spirulina at 50 mg/l (*T*_2_)	208.12 ± 0.89	243.87^d^ ± 6.19	255.28^a^ ± 0.59	283.75^a^ ± 11.7	28.30
Arsenic trioxide at 100 mg/l plus spirulina at 100mg/l (*T*_3_)	208 ± 0.92	235.25^a^ ± 3.66	222.37^c^ ± 0.59	260.35^a^ ± 9.44	23.50
Arsenic trioxide at 100 mg/l plus spirulina at 200 mg/l (*T*_4_)	207 ± 1.04	212.37^a^ ± 7.38	207.56^d^ ± 1.85	242.75^ad^ ± 4.03	15.90

Figure in mean.

SE: Standard error.

According to DMRT, column figures with or without superscripts do not change substantially.

Dates were estimated at the 99% level of significance (*p <* 0.01).

It was also shown that at a larger dose of spirulina (200 mg/l) in drinking water, the rise in biochemical parameters was minimal, implying that spirulina may have a protective function against arsenic-induced tissue damage to some extent. The specific reason for its protective effect in the recovery of tissue injury is unknown. Spirulina, on the other hand, is highly protein enriched, high in amino acids, iron, vitamin B complex, important fatty acids, and others responsible for maintaining good health. Our findings suggest that spirulina can help reduce elevated biochemical markers.

### Gross post-mortem lesions in chickens

Detailed post-mortem examinations of five sacrificed chickens from each group at every 7-days interval were performed up to 28 days after the experiment. The degree and extent of gross pathological lesions detected in various treated groups differed only in severity and depth, but no changes were identified in the control group. The characteristic pathologic defects were observed in the tissues and organs of birds. As poisoning progressed, enlarged liver and pinpoints and hemorrhages were found in chickens of group *T*_1_ on day 28. Similar lesions were found in groups *T*_2_, *T*_3_, and *T*_4_ but were moderate in nature. Infection with arsenic results in swollen, pale kidneys, with pinpoint hemorrhages in chickens of group *T*_1_ on day 28. On day 28, the *T*_1_ group shows lesions of the proventricular epithelium and colonic serosa followed by pervasive, degenerative hemorrhagic patches on the intestine’s mucosal surface. Myocardial hemorrhages were observed in group *T*_1_ on day 28. But in groups *T*_2_, *T*_3_, and *T*_4_, myocardial hemorrhages were moderate. In the brain and muscle, no observable lesions were found.

Similar to the current findings, the majority of the writers discovered pathological abnormalities in a variety of organs [23]. Significant occlusion in the visceral organs like the heart, liver, spleen, and kidney, deep purple erythema in the proventriculus and gizzard, and acute hemorrhagic enteritis were seen in arsenic-induced ducks [23]. However, no stomach lesions of this nature were seen in our research. Due to arsenic feedings, four organs, including the liver, kidney, gut, and heart, of birds were damaged in this study. Three doses of spirulina were not enough to prevent the lesions. The lesions were less severe in the chickens that were given arsenic and spirulina, which suggests that spirulina might help arsenic-caused lesions heal.

## Conclusion

The current findings held to account for arsenic’s deleterious effect on the body, causing ominous symptoms, decreased weight gain, changes in various hematological and biochemical parameters, and broody lesions. In that case, spirulina was proven to be the best in body weight gain in the broiler, comprised of the arsenic-induced diet. Henceforth, commercial feed formulations may add spirulina to overcome the toxic effects of arsenic in broilers.

## List of Abbreviations

ALT, alanine transferase; GDP, gross domestic product; LCD, liquid crystal display; ALP, alkaline phosphatase; AST, aspartate aminotransferase; DMRT, Duncan’s multiple range test; EDTA*,* ethylenediamine tetraacetic acid; FCR, feed conversion ratio; Hb, hemoglobin; PCV, packed cell volume; SGOT, serum glutamic oxaloacetic transaminase; SGPT, serum glutamate pyruvate transaminase; TEC, total erythrocyte count; TLC, total leukocyte count.
